# A Retrospective Analysis of Utilization Patterns and the National Accreditation Board for Hospitals and Healthcare Providers (NABH)-Mandated Blood Bank Quality Indicators at a Tertiary Care Hospital in South India

**DOI:** 10.7759/cureus.84876

**Published:** 2025-05-27

**Authors:** Sabari Priya, Sowmya Srinivasan, Karthikeyan V

**Affiliations:** 1 Transfusion Medicine, Mahatma Gandhi Medical College and Research Institute, Sri Balaji Vidyapeeth University, Puducherry, IND; 2 Pathology, Mahatma Gandhi Medical College and Research Institute, Sri Balaji Vidyapeeth University, Puducherry, IND

**Keywords:** analysis, benchmarkvalue, elective, nabh, preventive measures, quality indicators, root cause analysis, tat, tti, turnaround time

## Abstract

Background and objective

The objective of any blood transfusion service is not only to provide sufficient blood and blood components but also to supply zero-risk, efficient transfusion with minimal wastage through the implementation of a quality management system (QMS). This study aimed to analyze utilization patterns and monitor National Accreditation Board for Hospitals and Healthcare Providers (NABH)-mandated quality indicators (QIs) as performance tools related to blood transfusion services at a tertiary care hospital in South India.

Materials and methods

The study involved a retrospective cross-sectional analysis that spanned a year from August 1, 2023, to July 31, 2024, at the Department of Transfusion Medicine, Blood Center at the hospital. The information was gathered, and any deviation from the benchmark value was analyzed. The root cause analysis (RCA) of the deviation was performed, and corrective and preventive measures (CAPAs) were implemented

Results

There were about 2813 blood donations during the study period: 2641 (94%) donations by voluntary donors and 172 (6%) from replacement donors. The total number of blood and blood components prepared was 7950 units. The total number of crossmatches performed was 4530, and the total number of components transfused was 7705. The analysis of utilization patterns showed that the crossmatch/transfusion (C/T) ratio was 0.8, the transfusion probability was 100%, and the transfusion index (TI) was 1.7. All these indices revealed that there is efficient utilization of blood components in our hospital. The analysis of NABH-mandated key performance indicators (KPIs) revealed the following findings: rate of transfusion-transmitted infections (TTIs): 31 (1.1%); adverse transfusion reaction rate (ATRR): 20 (0.3%); percentage of components issued: 99.9%; adverse donor reaction (ADR) rate: six (0.2%); donor deferral rate (DDR): 192 (6.4%); wastage rate among units issued: 17 (0.2%); and wastage rate among various components - whole blood (WB): 0%, packed red blood cells (PRBCs): 0.1%, fresh frozen plasma (FFP): 0.3%, platelets: 20%, and cryoprecipitate: 0%. The turnaround time (TAT) for elective cases was 30 minutes, and that for emergency cases was also 30 minutes.

Conclusions

The regular analysis of pattern utilization of blood and blood components and monitoring of NABH-mandated KPIs at a hospital will help identify problems, perform root cause analysis, which facilitates necessary corrective and preventive actions, thereby paving the way for promoting high standards of quality in all aspects of patient care and hospital transfusion services.

## Introduction

Blood transfusion services play an important role in the resuscitation and management of patients. It forms an integral and essential part of hospital services [1]. The requirement for blood continues to exceed the volume collected by blood centers. Hence, efficient use of various blood and blood components with high quality and minimum wastage is a key goal of the blood utilization management system. Various studies have shown that there is gross overordering of various blood and blood components, causing financial burden to both the patient and the hospital, as well as loss of precious time and manpower [2]. Periodic review and monitoring usage of various blood and blood components will enable us to analyze the blood utilization pattern at hospitals.

The goal of any blood transfusion service involves not merely meeting demands but also providing zero-risk and efficient transfusion with minimal wastage, which can be achieved by implementing a quality management system (QMS) [3]. QMS can be monitored with the help of key performance indicators (KPIs) known as quality indicators (QIs) [4]. The American Association of Blood Banks (AABB) defines quality indicators as specific performance measurements designed to monitor one or more processes and are useful for evaluating service demands, production, adequacy of personnel, inventory control, and process stability [5].

Regular monitoring of QIs, performing root cause analysis (RCA), and taking corrective and preventive actions (CAPAs) will aid QMS in the robust performance of transfusion services. Many blood centers have been accredited by the National Accreditation Board for Hospitals and Healthcare Providers (NABH) [6]. NABH conducts assessments focused on KPIs to safeguard the integrity and quality of blood products [7]. Establishing and meeting the criteria of such quality indicators has become a necessity for establishing blood safety from the donor’s vein to the patient’s vein [8]. The present study aims to analyze the utilization pattern and monitor NABH-mandated QIs as performance tools related to blood transfusion services.

## Materials and methods

This was a retrospective cross-sectional study carried out over one year at the Department of Transfusion Medicine and Blood Center at a tertiary care hospital in South India. The ethical clearance to conduct the study was obtained from the Institutional Human Ethics Committee (project no MGMCRI/2025/01/04/IHEC/36). The Study duration was from August 1, 2023, to July 31, 2024. Blood center records of details of all blood donors who donated blood during the study period, details of all transfusion recipients, requests received, components crossmatched, components issued, and details of blood components discarded were assessed. Transfusion requests and blood components issued to outside hospitals and incomplete records were excluded from the study.

Sampling technique**

A universal sampling technique was employed, and relevant records of blood donors and transfusion recipients were analyzed retrospectively.

Study procedure

The details collected retrospectively from blood bank records of both blood donors and transfusion recipients were as follows

Blood Donors

The details collected from donor records included total number of donors counseled, number of blood donors who donated blood, number of donors deferred from blood donation, reasons for deferral, age, sex, blood group of donors, type of donors (whether voluntary or replacement), first time/repeat donors, number of donors with adverse donor reaction (ADR) rate, number of transfusion transmitted infections (TTIs: HIV, HBsAg, HCV, malaria, syphilis), and reactive donors.

Transfusion Recipients

The details collected from transfusion recipients’ records included total number of patients who requested blood and blood components, type of blood and blood components requested, number and type of blood and blood components transfused during the study period, age, sex, blood group, diagnosis, number of blood and blood components wasted among those issued and components discarded on shelf due to expiry, breakage, hemolysis, turnaround time (TAT) for blood and blood components crossmatched and reserved for both elective and emergency cases, adverse transfusion reactions, percentage of quality control (QC) failure among various components, ratio of number of components crossmatched to number of components transfused, number of patients crossmatched, and the number of patients transfused 

Parameters Studied and Study Tools

Two separate data collection sheets for data regarding blood donors and blood transfusion recipients were recorded in Excel, and the following parameters were analyzed.

QIs and the formula that were used to assess the appropriateness of utilization of blood and blood components are as follows [9]

(1) Crossmatch-to-transfusion (C/T) ratio: The C/T ratio is defined as the number of units crossmatched to the number of units transfused; a value of 2.5 and below is considered significant blood usage.

(2) Transfusion probability (TI%): This is defined as the number of patients transfused to the number of patients crossmatched; a value of 30% and above is considered significant blood usage.

(3) Transfusion index (TI): TI is defined as the number of units transfused to the number of patients crossmatched. A value of more than 0.5 is considered indicative of significant blood usage.

KPIs or QIs proposed by NABH [10] that were studied were as follows: percentage of TTIs, Percentage of adverse transfusion reaction rate (ATRR), wastage rate, TAT for blood issues, percentage of ADR rate, component QC failure for each component, donor deferral rate (DDR), and percentage of component issues.

The formulae for the above-defined KPIs are described in Table 1.

**Table 1:** Key performance indicators described by NABH with formulas ADR: adverse donor reaction; ATRR: adverse transfusion reaction rate; DDR: donor deferral rate; HBV: hepatitis B virus; HCV: hepatitis C virus; HIV: human immunodeficiency virus; NABH: National Accreditation Board for Hospitals and Healthcare Providers; QC: quality control; TAT: turnaround time; TTI: transfusion-transmitted infections

SI no.	Parameter	Formula
1.	TTI%	Combined TTI cases (HCV +HIV + HBV + syphilis + malaria)/total number of donors x 100
2.	ATRR%	Number of adverse transfusion reactions/total number of blood and blood components issued x 100
3.	% component	Total component issues/total whole blood + component issues x 100
4.	ADR rate%	Total number of donors experiencing donor reaction/total number of donors x 100
5.	DDR	Number of donor deferrals/total number of donations + total number of deferrals x 100
6.	Component QC failures for each component	Number of components QC failure/total number of components tested x 100
7.	Wastage rate % (issued units)	Number of blood and blood components discarded/total number of blood and blood components issued x 100
8.	Wastage rate% (on-shelf)	Number of blood and blood components discarded/total number of blood and blood components on shelf x 100
9.	TAT for blood issues (elective cases)	Sum of time taken/total number of blood and blood components crossmatched/reserved
10.	TAT for blood issues (emergency cases	Sum of time taken/total number of blood and blood components crossmatched/reserved

Values obtained from the above formulas (Table 1) were compared with the standard benchmark value [7]. RCA for the deviation of each of the QIs from the benchmark value was conducted, and necessary CAPAs were implemented.

Statistical analysis

The data collected were recorded in Microsoft Excel and analyzed. Quantitative data were summarized in terms of mean and standard deviation (SD). Qualitative data were summarized in terms of frequency and percentage.

## Results

During the one-year study period, 2813 blood donations were documented, comprising 2641 (94%) donations by voluntary donors and 172 (6%) by replacement donors. Of these, 2794 (99%) were made by male donors, while 19 (1%) were made by female donors. Out of 3005 blood donors who were counseled, 192 (6.4%) donors were deferred from blood donation due to various reasons. Six donors developed an adverse donor reaction. The total number of blood and blood components prepared during the study period was 7950 units, which included four (0.05%) whole blood (WB), 2797 (35.1%) packed red blood cells (PRBCs), 2291 (28.8%) platelets, 2763 (34.8%) fresh frozen plasma (FFP), and 95 (11.9%) of cryoprecipitate. The total number of blood requests received from various departments was 6003, which included 1075 requests for elective cases and 4928 emergency requests. The total number of blood and blood components transfused was 7705, which included four (0.1%) WB, 2746 (35.6%) PRBCs, 3183 (41.3%) units of FFP, 1678 (21.8%) units of platelets, and 94(1.2%) units of cryoprecipitate.

In the present study total number of units crossmatched was 4530, the total number of units transfused was 5933, the total number of patients transfused was 2908, and the total number of patients crossmatched was 2900. This data were used to calculate the blood utilization patterns at our hospital with blood indices (Table 2), which demonstrated significant usage.

**Table 2:** Patterns of blood utilization observed

Sl no.	Blood transfusion indicators	Value	Utilization status
1.	Crossmatch-to-transfusion (C/T) ratio	0.8 (cutoff: <2.5)	Significant blood usage
2.	Transfusion probability (TP%)	100% (cut-off: >30%)	Significant blood usage
3.	Transfusion index (TI)	2.0 (cut-off: >0.5)	Significant blood usage

Our analysis of NABH-mandated blood bank QIs from the data collected retrospectively for one year showed that the overall TTI rate was 31 (1.1%), the total percentage of blood and blood components issued was 99.9%, and the ATRR reported was 20 (0.3%). The overall wastage rate of various blood components was as follows: WB: 0%, packed cells: five (0.1%), platelets: 589 (20%), FFP: 32 (0.3%), and cryoprecipitate: 0%. Wastage rate due to return of unused blood and blood components was 17 (0.1%). The ADR rate was six (0.2 %), and the DDR during the study period was 192 (6.4%). The percentage of QC failure rate for various components was as follows: WB: 0%, PRBC: 11 (8%), FFP: 3 (6%), platelets: 32 (30%), and cryoprecipitate: 0%. The overall TAT for elective cases was 30 minutes, and that for emergency cases was also 30 minutes. The overall outcomes of QIs during the study period are summarized in Table 3 and illustrated in the images that follow.

**Table 3:** Summary of blood bank quality indicators ADR: adverse donor reaction; ATRR: adverse transfusion reaction rate; BV: benchmark value; Cryo: cryoprecipitate; DDR: donor deferral rate; FFP: fresh frozen plasma; HBV: hepatitis B virus; HCV: hepatitis C virus; HIV: human immunodeficiency virus; Plts: platelets; PRBCs: packed red blood cells; QC: quality control; TAT: turnaround time; WB: whole blood

SI no.	Quality indicator	Calculation	Overall rate	BV [7]	BV achieved: Yes/No
1.	TTI% = combined TTI cases (HCV +HIV + HBV + syphilis + malaria)/total number of donors x 100	HBV: 6 (0.2%), HIV: 3 (0.1%), HCV: 3 (0.1%), syphilis: 18 (0.6%), malaria: 1 (0.03%)	1.1%	<4%	Yes
2.	ATTR = number of adverse transfusion reactions/total number of blood and blood components issued x 100	20/7705 x 100	0.3%	<2%	Yes
3.	Percentage of component issues = total component issues/total whole blood + component issues x 100	7701/7705 x 100	99.9%	100%	No
4.	ADR rate = total number of donors experiencing donor reaction/total number of donors x 100	6/2813 x 100	0.2%	<2%	Yes
5.	DDR = number of donor deferrals/total number of donations + total number of deferrals x 100	192/3005 x 100	6.4%	No set benchmark value	No set BV
6.	Component QC failures for each component = number of component QC failures/total number of components tested x 100	PRBC: 11/135 x 100, FFP: 3/49 x 100, Plts: 32/107 x 100	PRBC: 8%, FFP: 6%. Plts: 30%,	75% should conform to QC standards	Plts' QC failure rate was high
7.	Wastage rate% (issued units) = number of blood and blood components discarded/total number of blood and blood components issued x 100	17/7705 x 100	0.2%	<1%	Yes
8.	Wastage rate% (on-shelf) = number of blood and blood components discarded/total number of blood and blood components on shelf x 100	WB: 0/4 x 100, PRBC: 5/4489 x 100, FFP: 32/8175 x 100, Plts: 589/2841 x 100, Cryo: 0/319 x 100	WB: 0%, PRBC: 0.1%, FFP: 0.3%, Plts: 20%, Cryo: 0%	PRBC: <1%, Plts: <22%, FFP + Cryo: <1%	Yes
9.	TAT for blood issues (elective cases) = sum of time taken/total number of blood and blood components crossmatched/reserved	355.82/1076 = 0.3	30 minutes	Self-set parameter (30-45 minutes)	Yes
10.	TAT for blood issues (emergency cases) = sum of time taken/total number of blood and blood components crossmatched/reserved	1519/4928 = 0.3	30 minutes	Self-set parameter (30-45 minutes)	Yes

**Figure 1:**
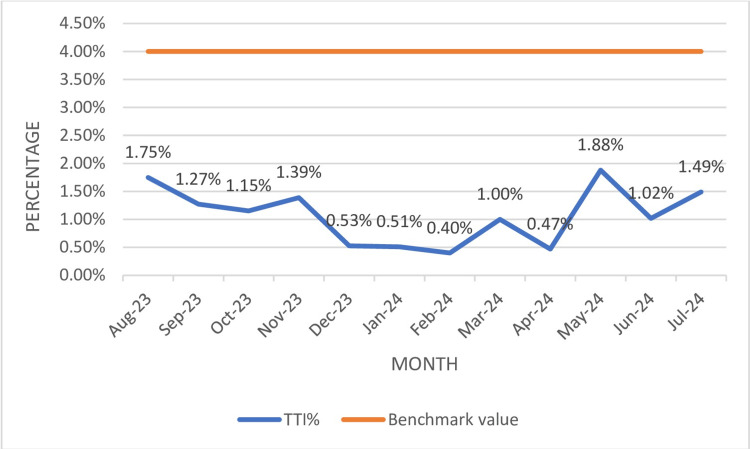
TTI rate data TTI: transfusion-transmitted infections

The overall TTI% during the study period was 1.1% (Table 3). TTI% was much below the benchmark value of 4%. The highest TTI% recorded was in May 2024, while the lowest TTI% recorded was in February 2024, as illustrated in Figure 1

**Figure 2:**
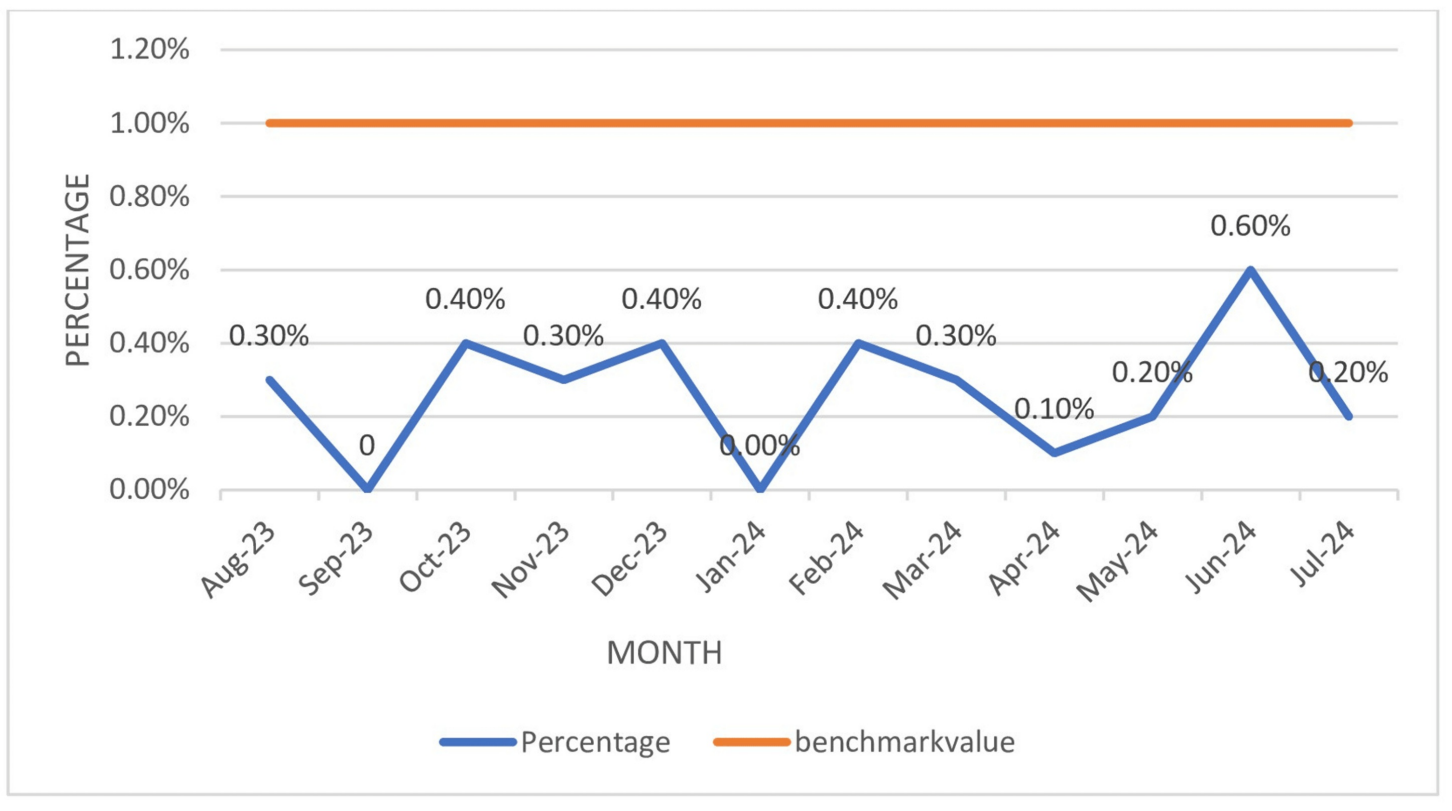
Percentage of ATRR (ATRR%) ATRR: adverse transfusion reaction rate

The overall ATRR% recorded during the study period was 0.3% (Table 3), below the benchmark value of 1%. The highest percentage of ATRR was recorded in June 2024 (0.6%), as illustrated in Figure 2.

**Figure 3:**
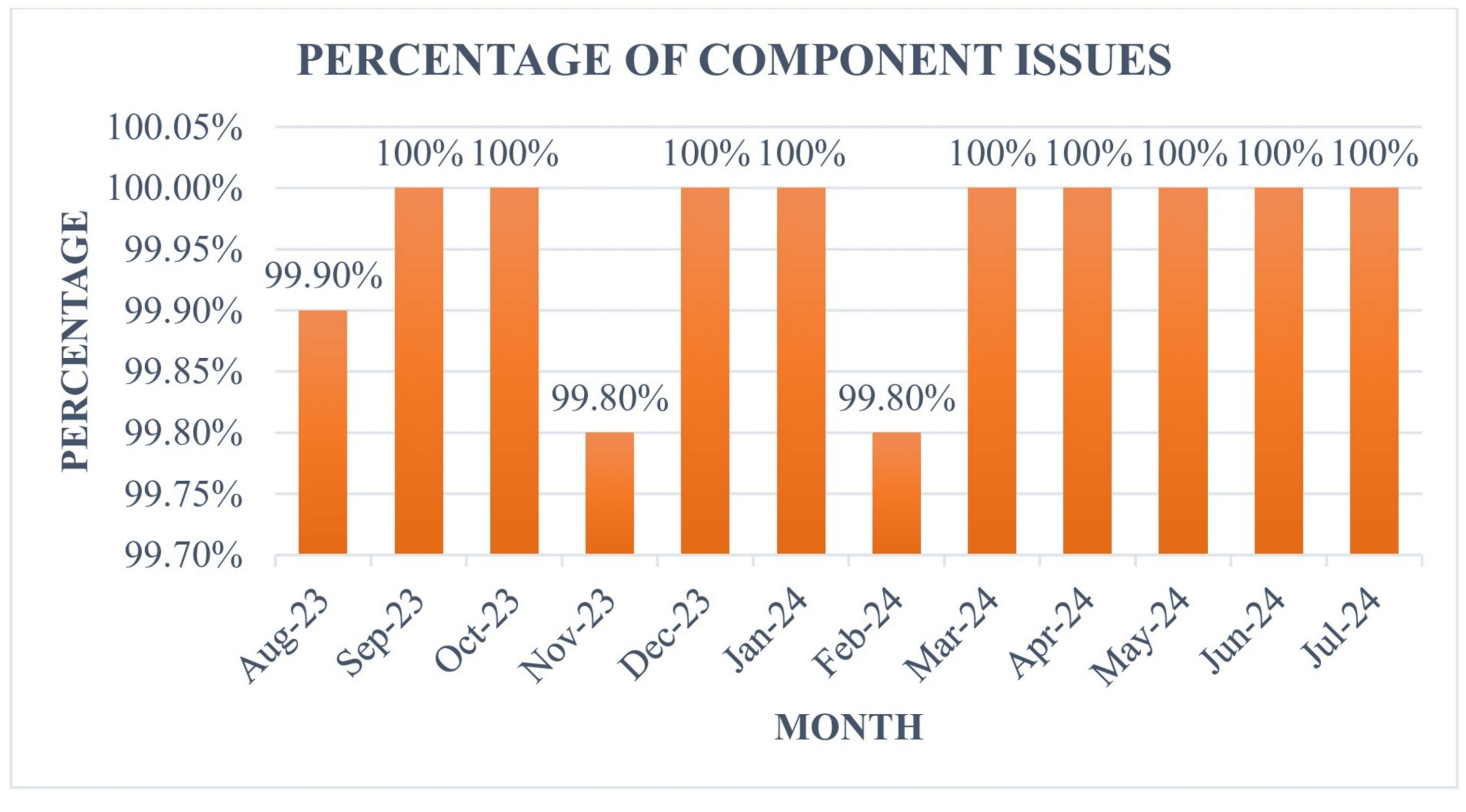
Percentage of component issues

The overall percentage of component issues during the study period was 99.9% (Table 3), below the benchmark value of 100%. Benchmark value of 100% was achieved in most months, as illustrated in Figure 3.

**Figure 4:**
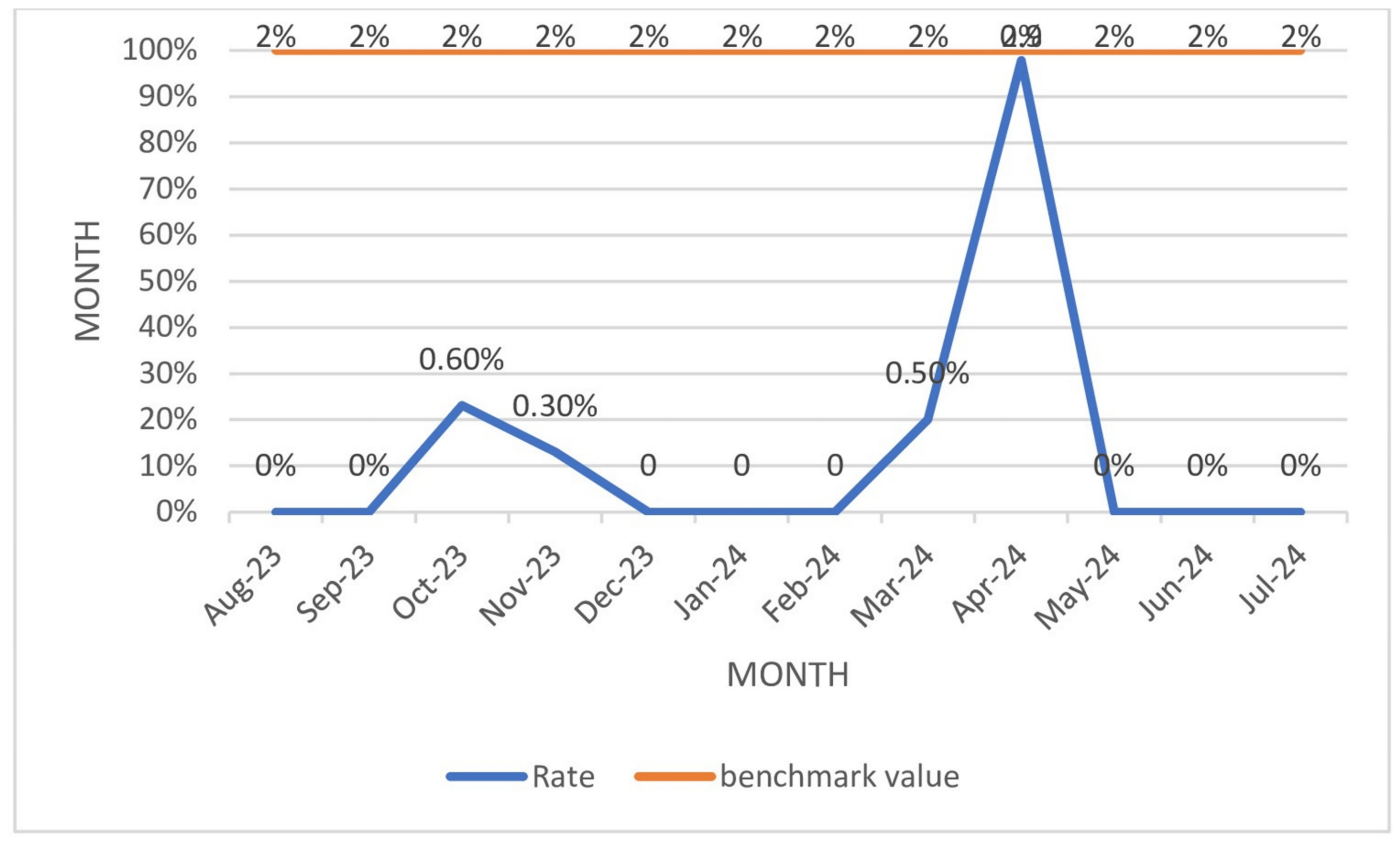
Percentage of ADR rate (ADRR%) ADR: adverse donor reaction

ADR rate % recorded during the study period was 0.2% (Table 3), much below the benchmark value of 2%. The highest deferral rate (0.9%) was recorded in April 2024, as illustrated in Figure 4.

**Figure 5:**
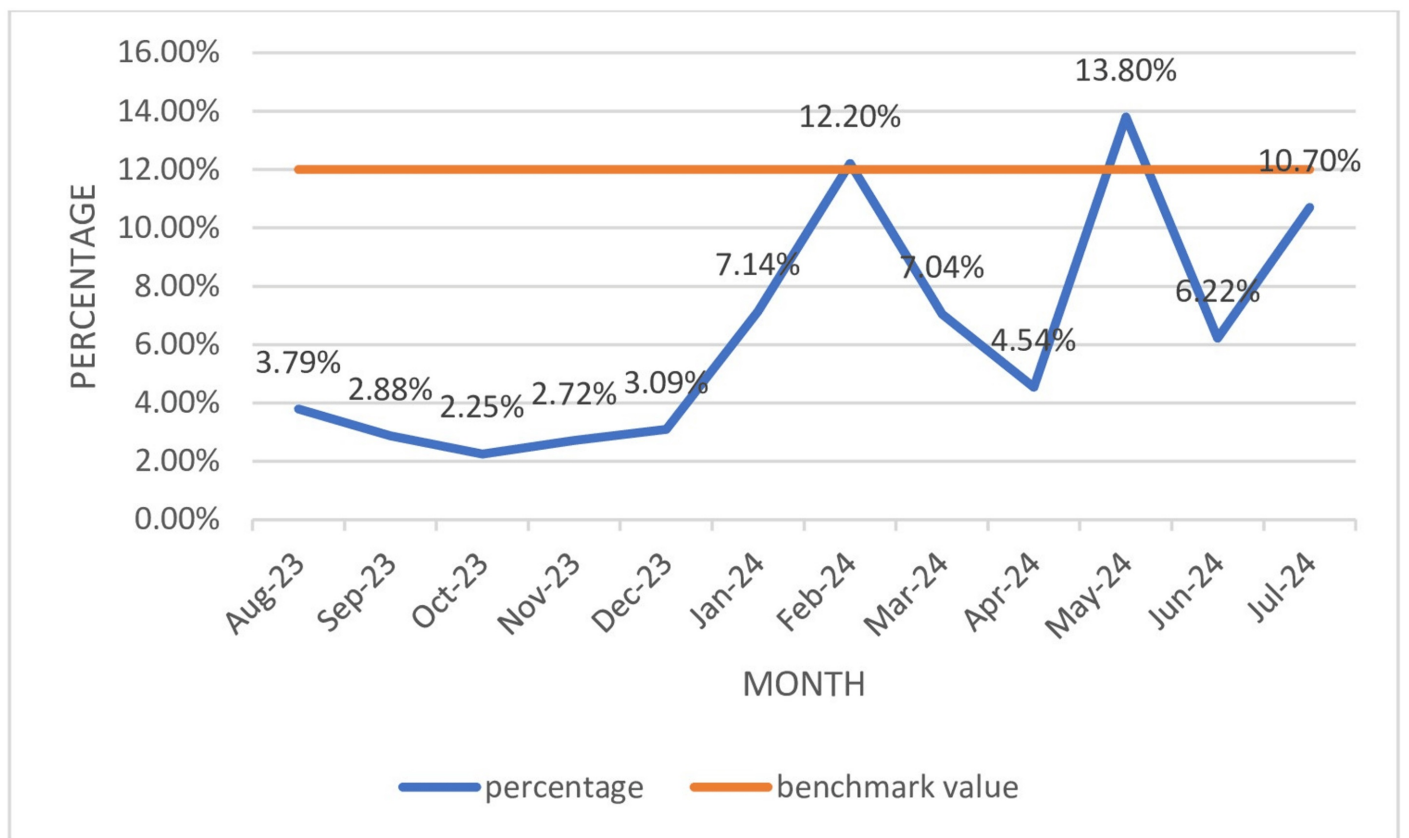
Percentage of DDR (DDR%) DDR: donor deferral rate

The overall DDR% observed during the study period was 6.4% (Table 3), much below the benchmark value of 12%. The DDR was 12.2% and 13.8% in February 2024 and May 2024, respectively, much above the benchmark value (Figure 5).

**Figure 6:**
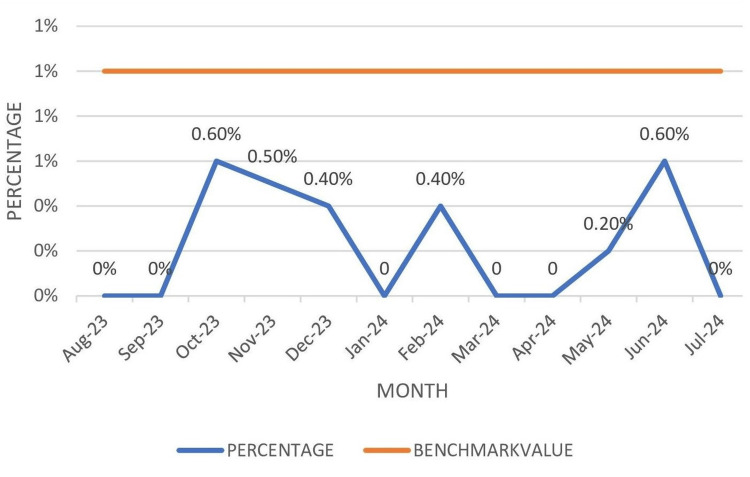
Wastage rate among units issued

The QC failure rate recorded for various components (Table 3) during the study period was 8% for PRBCs, 6% for FFP, and 30% for platelets. Overall wastage rate among various units issued recorded during the study period was 0.2% (Table 3), below the benchmark value of 1%, as illustrated in Figure 6.

**Figure 7:**
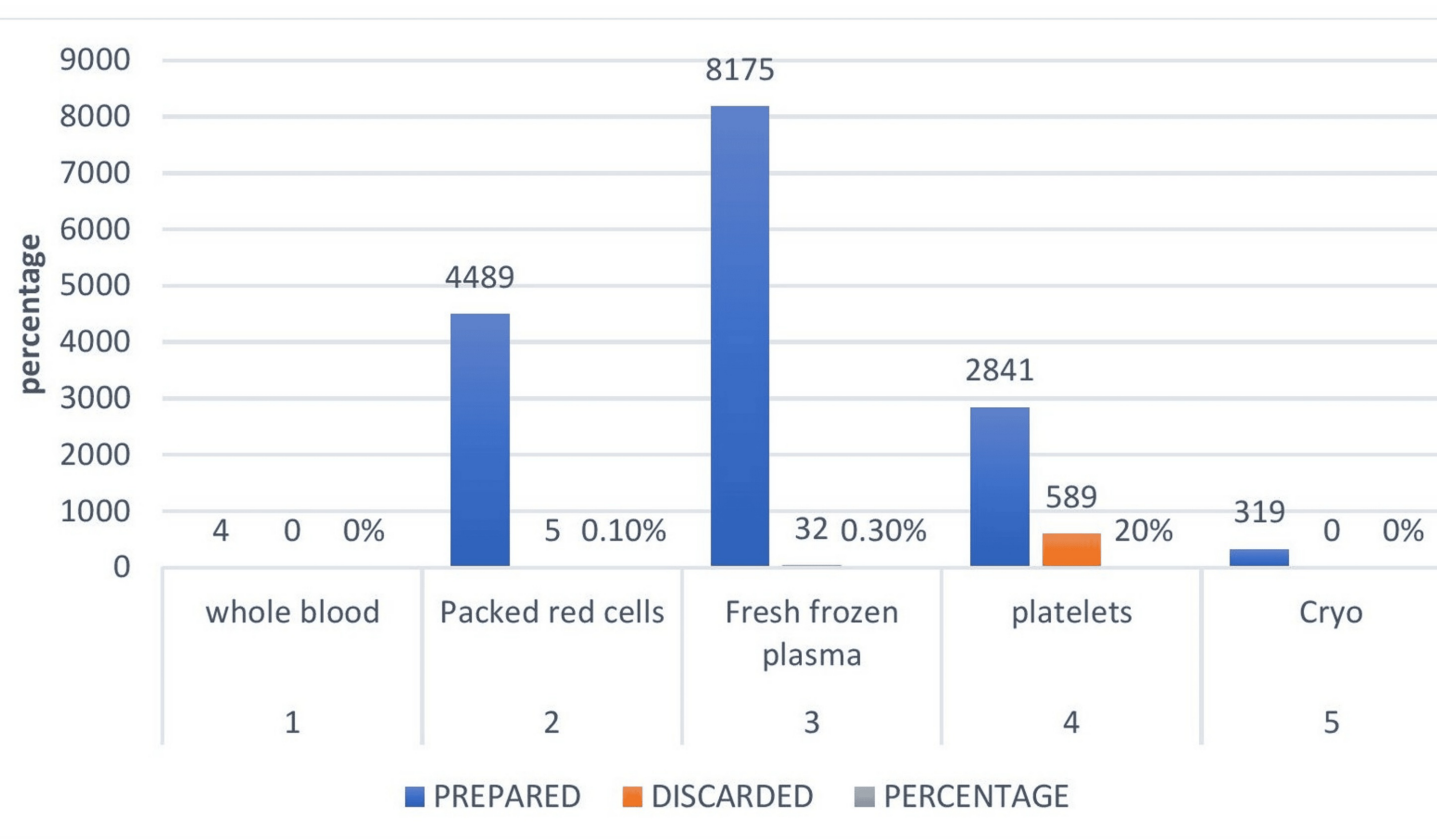
Wastage rate of various blood components on-shelf Benchmark value: PRBC: <1%; Plts: <22%; FFP + Cryo: <1% Cryo: cryoprecipitate; FFP: fresh frozen plasma; Plts: platelets; PRBCs: packed red blood cells

The overall wastage rate of various components (Table 3) on-shelf during the study period was as follows: WB: 0%, PRBCs: 0.1%, FFP: 0.3%, platelets: 20%, and cryoprecipitate: 0%. The discard rate for various blood components was within the benchmark value, as illustrated in Figure 7.

**Figure 8:**
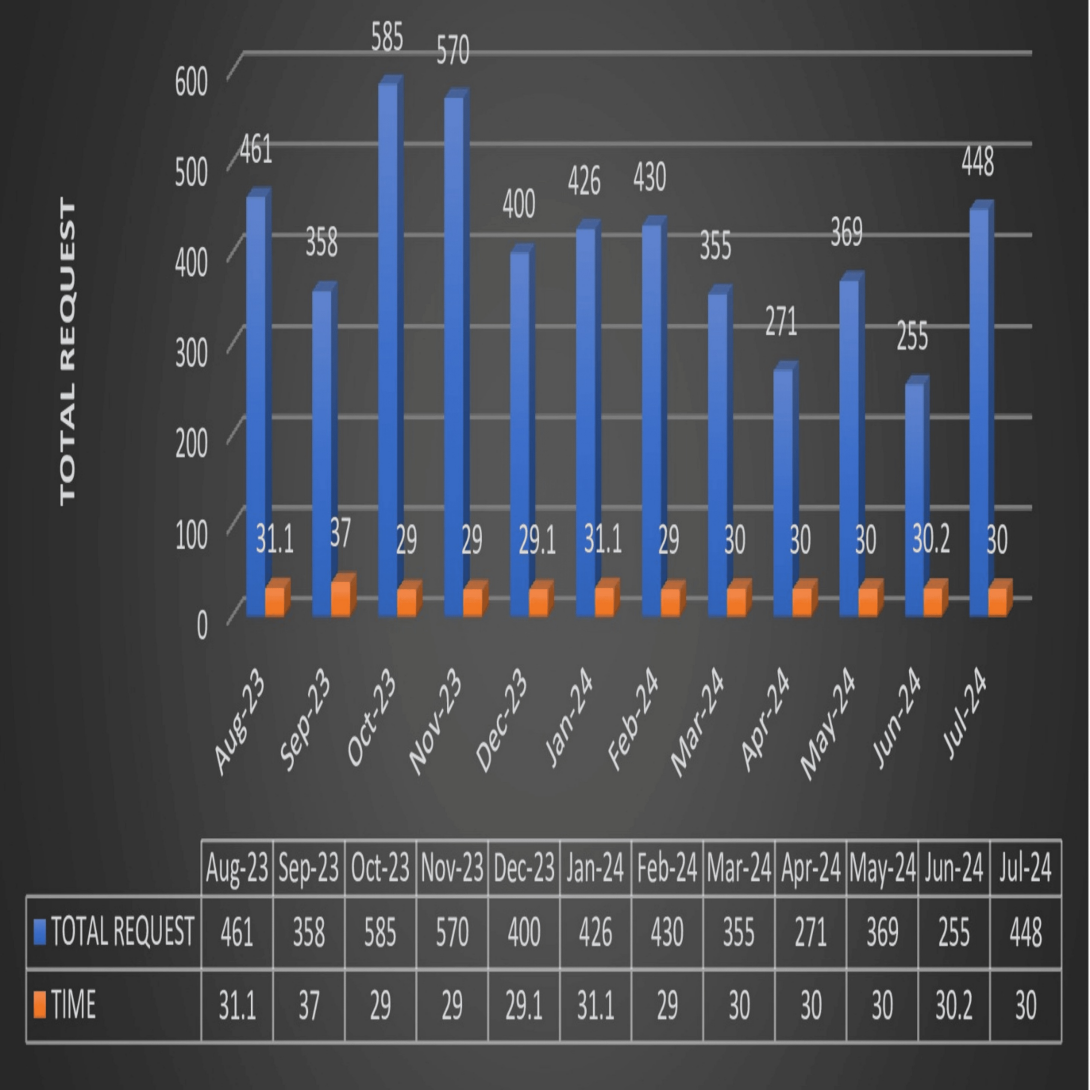
TTA for emergency cases TTA: turnaround time

The TTA time for emergency cases during the study period was 30 minutes (Table 3), as illustrated in Figure 8.

**Figure 9:**
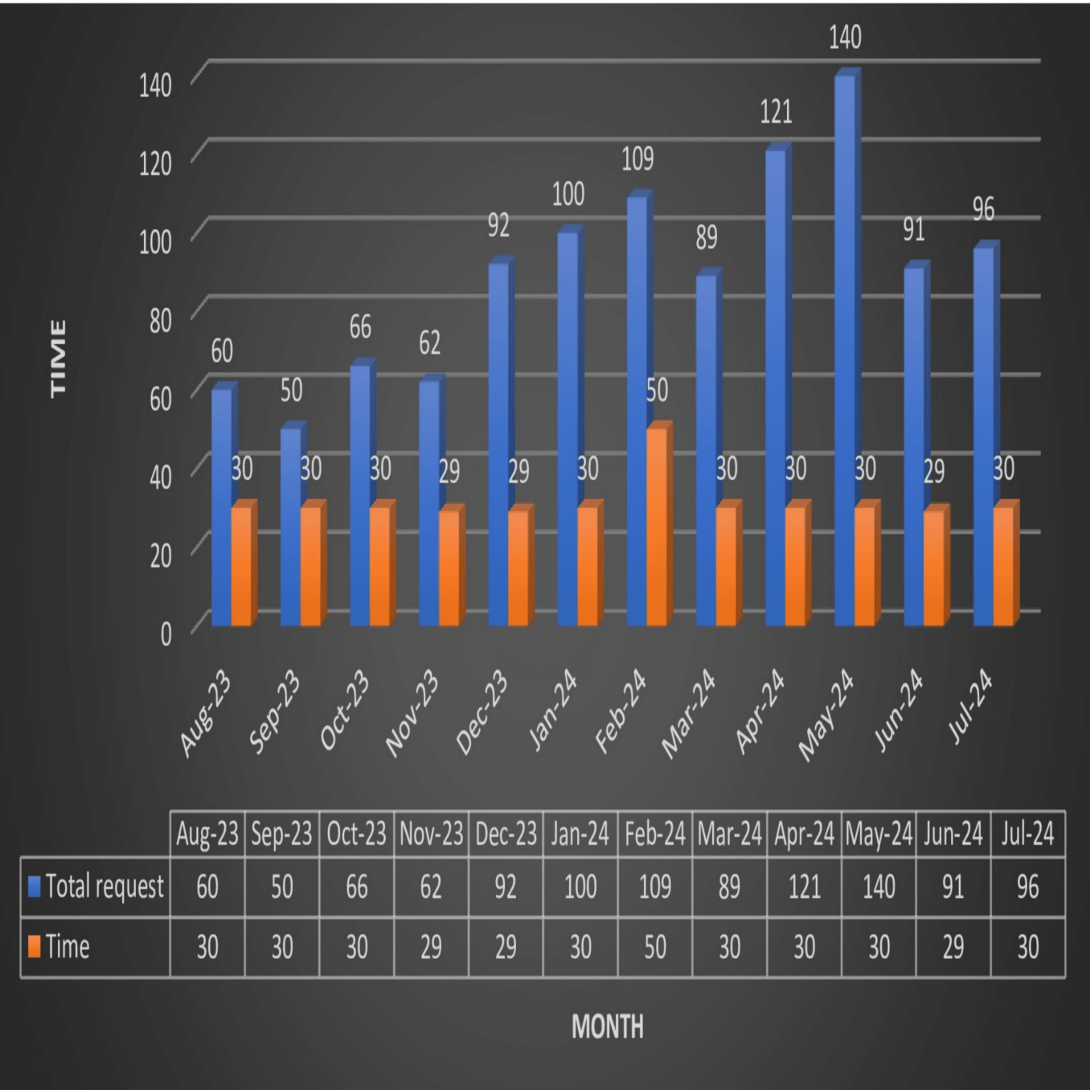
TTA for elective cases TTA: turnaround time

Overall TTA for elective cases documented during the study period was 30 minutes, Table 3, as illustrated in Figure 9.

## Discussion

Blood and blood components are a very precious, indispensable, expensive, and scarce resource. A well-structured and organized blood transfusion service contributes to better healthcare for patients in need. Various strategies have been developed to ensure this, which include guidelines, policies, training programs, CME (continued medical education), webinars, quiz competitions, monitoring, and auditing of transfusion practices to prevent inappropriate use of blood and blood components [11]. It is very important and essential to have a well-structured and organized quality assurance program to ensure safe and effective transfusion service [12]. QIs have a vital part to play in maintaining performance and ensuring safety and integrity for the recipient and the donor. QI data can be used for continuous measurement, identifying problems, performing RCA, implementing corrective action, developing a quality improvement strategy (preventive action), reporting, and seeking opportunities for improvement [8].

In light of this, the current retrospective study involved monitoring quality indicators, which included C/T ratio, TP, and TI. Efficient use of blood and its components with high quality and minimum wastage is an important goal of the blood utilization management system [13]. The pattern of blood utilization varies from hospital to hospital. The various QIs that have been identified by WHO are the C/T ratio, TP (%), and TI [14].

We observed that our hospital's overall C/T ratio was 0.8%. Performing unnecessary crossmatches and reserving blood and blood components results in unnecessary wastage of blood bank resources like manpower and reagents. Appropriate use of blood and blood components is an extremely cost-effective practice [15]. According to our blood bank SOP (standard operating procedure), crossmatching is performed only for PRBCs and FFP before issuing. The C/T ratio of 0.8 in this study is much lower than that compared to various studies in the literature: Devi et al. [11], Anigbogu et al. [16], Belayneh et al. [17], Kuchhal et al. [18], Kaur et al. [19], Yasmeen et al. [20], Raghuwanshi et al. [21], Zewdie et al. [22], Yasmeen et al. [23], and Paudel et al. [24] (Table 5). This indicates significant blood usage as C/T must be ideally less than 2.5 [9]. At our institution, a policy was implemented stating that whenever a blood requisition form was submitted to blood bank, only type and screen (blood grouping, antibody screening) would be performed; if blood is required, a blood release form (having information regarding patient details, type of component required, and doctor’s signature) must be given for both elective and emergency cases. This prevents unnecessary crossmatches and thereby conserves manpower and resources to a great extent.

Mead et al. have described the concept of transfusion probability (TP%) [25]. In the present study, the transfusion probability was 100%; a value of 30% or more has been suggested to indicate efficient blood usage [26]. The transfusion probability of the current study is much higher when compared to other studies in the literature (Table 5). TI shows how many blood units are crossmatched appropriately. An effective usage of blood is indicated by a value of 0.5 or above [27]. In the present study, TI was reported to be 2.0, which is much higher when compared to other studies in the literature (Table 5). Overall inference of the quality indicators data on utilization pattern of blood and blood components of our hospital is within acceptable reference limits [9]. Hospital Transfusion committee meeting, clinical audits, and training program can be used to frame MSBOS (Maximum Surgical Blood Ordering Schedule), which can cut down unnecessary blood bank expenditure and conserve blood bank resources and funds to a great extent [27].

**Table 4:** Comparison of utilization pattern of blood components among various studies in the literature C/T ratio: crossmatch-to-transfusion ratio; TI: transfusion index; TP: transfusion probability

SI no.	Study	C/T ratio	TI	TP%
1.	Devi et al. [11]	1.02	0.97	97.2
2.	Anigbogu et al. [16]	1.3	1.1	71
3.	Belayneh et al. [17]	2.3	0.77	47
4.	Kuchhal et al. [18]	1.8	0.5	61.7
5.	Kaur et al. [19]	1.57	1.18	79.0
6.	Yasmeen et al. [20]	1.12	0.88	88.8
7.	Raghuwanshi et al. [21]	6.31	0.65	51.62
8.	Zewdie et al. [22]	7.6	0.29	15.3
9.	Yasmeen et al. [23]	1.1	1.1	68.1
10.	Paudel et al. [24]	2.7	37.5	0.42
11.	Present study	0.8	2.0	100

To get a complete idea regarding the quality control of the blood banking process, we decided to evaluate our findings based on various mandatory quality indicators as per NABH.

The overall TTI% (Table 3, Figure 1) during the study period was 1.1%, which was much below the NABH benchmark value of 4%. The TTI% rate of 1.1% (HIV: 0.1%, HBV: 0.2%, HCV: 0.1%, syphilis: 0.6%, and malaria: 0.03%.) in the current study is similar to the TTI% reported by Mukerjee et al. (1.8%) and Das et al. (1.87%) [28, 29]. The TTI% % rate varies among different studies, ranging from 0.6% to 4.05% [6, 30-32]. Differences among various studies could be due to varied demographic profiles. Among the various TTI rates reported in the current study, the highest seroprevalence was reported for syphilis (18, 0.6%), followed by HBV (6, 0.2%), which contrast with the study by Varshney et al. [8] in which highest seroprevalence was reported for HBsAg, followed by syphilis and HCV. The highest seroprevalence reported for syphilis in the current study emphasizes the need to improve and strengthen pre-donation counseling, deferring donors with high-risk behaviour. The second highest seroprevalence reported for HBV emphasizes the need to increase the voluntary blood donation pool over replacement donors, the need to conduct more voluntary blood donation awareness programs targeting the young population, and educating donors with high-risk behavior for self-deferral. Also, more sensitive testing strategies like chemiluminescence can be implemented.

The ATRR% (Table 3, Figure 2) during the study period was 0.3%, which aligns with the findings of Mukerjee et al. (0.3%) [28]: lower than the NABH benchmark value of 2%, but slightly higher than those reported in other studies in the literature, ranging from 0.14% to 0.18% [31,33-34]. The most common transfusion reaction reported was allergic transfusion reaction, followed by febrile non-hemolytic transfusion reaction. Leukocyte-reduced blood products can be used to reduce the incidence of febrile transfusion reactions. Our institute has enrolled with the National Hemovigilance Program of India. Avoidance of pre-medication and regular education regarding reporting of transfusion reactions among healthcare professionals can be initiated.

The percentage of component issues (Table 3, Figure 3) during the study period was 99.9%, which is below the NABH benchmark value of 100%. As a part of our blood bank SOP and protocol, only blood components were issued; however, during the study period of one year, there were three cases, which included one case of neonatal exchange transfusion, one trauma, and a patient with gastrointestinal bleeding who required fresh whole blood. The percentage of component issues in the current study is comparable to those reported in various studies in the literature, ranging from 95.6 to 100% [7].

The ADR rate (Table 3, Figure 4) in the current study was 0.2%, comparable to the donor reaction rate reported by Hariharan et al. (0.58%) [31], Kumar et al. (0.93%) [35], and but lesser than the reaction rates reported by other studies in the literature [36]. Strict adherence to donor screening strategies, including details of last food intake, alcohol, travel history, pre-donation and post-donation counseling, providing at least 50 ml of fluids before blood donation, fewer number of female donors, and higher number of repeat donors could be the reason for the fewer donor reactions found in the current study.

DDR (Table 3, Figure 5) during the current study period was 6.4%, which aligns with lower deferral rates of 5.12% [37], 9.3% [8], and 8.99% [31] reported in some studies, but contrasting with higher deferral rates of 14.05% [7], 11.6% [38], and 12.4% [39] reported in others. Different deferral rates among various studies in the literature could be due to varying demographic profiles among the donor population. In the current study, the most common reasons for deferral were low hemoglobin, high blood pressure, inadequate sleep, alcohol intake, dental extraction, etc. Many male donors were deferred due to low hemoglobin, especially repeat donors; this could be due to more frequent repeat donations and inadequate nutrition, which emphasizes the need to screen blood donors, especially repeat donors, not only for hemoglobin but also serum ferritin. Other strategies to decrease DDR include conducting awareness programs, CMEs among college students and rural areas, and modifications and rationalization of donor deferral criteria. Also, counseling of blood donors can help reduce DDr and increase the donor return rate to a great extent.

In the present study, the QC (Table 3) failure rate was 8% for packed red cells, 6% for fresh frozen plasma, and 30 % for platelets. The QC failure rate of platelets was much above the NABH benchmark value. Root cause analysis for platelet QC failure was performed to help understand shortcomings like improper sample collection, delay in processing, sample not mixed properly, improper centrifugation, and improper storage. Regular training and intradepartmental training classes can be conducted for blood bank technicians, apart from ensuring the recruitment of a quality manager and implementation of the buffy coat method of preparation of blood components.

In this study, the wastage rate (Table 3, Figure 6) due to the return of blood and blood components among units issued was 0.2%, which was much below the NABH benchmark value of 1%. Wastage rate in our study was less when compared to other studies in the literature, which ranged from 0.1% to 10% [40]. In our institute, a policy for returning unused blood and blood components within 30 minutes was implemented, and an orientation and sensitization program was conducted targeting clinicians, nurses, students, and interns. For every return that happened beyond 30 minutes, a one-to-one interaction was conducted with the concerned faculty and people involved, starting from ordering transfusions, an event report was submitted to PSC (patient safety committee) and QCC (quality control committee). Monthly data were presented during mortality meetings.

In the present study, the wastage rate (Table 3, Figure 7) of various blood and blood components was as follows: WB 0%, PRBCs: 0.1%, FFP + cryoprecipitate: 0.3%, and platelets: 20%. The discard rate of various components was below the NABH benchmark value, and platelets were the most common component discarded due to expiry. The discard rate was similar to the findings of Rajkumar et al. [7] and Varshney et al. [8]. Though FIFO (First in first out) policy was followed and platelets are meant to be prepared only on demand, since the preparation is time consuming and depends on daily turnover of donors, a buffer stock of platelets is necessary to be maintained to meet emergencies (dengue, disseminated intravascular coagulation, massive transfusion) which, if not used, leads to wastage. The second most common component discarded was FFP, the discard rate of which aligned with that reported by Rajkumar et al. [7]. 

The policy followed at our Blood Center dictates that whenever a crossmatch requisition form and sample are submitted, only type and screen will be performed; if blood and blood components are required, a blood release form (having information regarding patient details, type of component required, and doctor’s signature) must be submitted. This avoids unnecessary crossmatches being performed, saving valuable resources, manpower, and time to a great extent. TAT (Table 3, Figures 8, 9) for the issue of blood and blood components for both elective and emergency cases is calculated from the time the blood release form is received till crossmatches are performed and reserved. In the current study, TAT for elective cases was 30 minutes, much lower compared to that reported in various studies in the literature [8]. TAT for emergency cases was 30 minutes, as in various studies in the literature [8]. In February 2024, the TAT for elective cases recorded was 50 minutes. TAT for emergency cases reported for August 2023 was 31.1, while it was 31.1 in January 2024. RCA of such delays revealed insufficient manpower, multiple components ordered, changes in the choice of components, untested units, improperly filled forms, and insufficient samples. Steps were taken to recruit extra technicians and conduct orientation classes for all regarding the rational use of blood and blood components; proper protocol for sample collection was implemented.

Limitations

The study has a few limitations. C/T ratio, transfusion probability, and transfusion index could not be calculated department-wide. As the study was conducted at a single tertiary care hospital in South India, the generalization and adaptability of its findings for other regions are limited. The retrospective design of the study and reliance on blood bank records can lead to information bias. The study could also have been done prospectively after corrective and preventive measures were implemented.

## Conclusions

This retrospective study involved monitoring of utilization patterns and NABH-mandated quality indicators, which we conducted as a team involving clinicians, interns, students, and blood bank staff. Its findings have helped us to understand and identify areas that need improvement and modifications in methodologies, protocols, policies to be framed, and guidelines to be followed. It has enabled us to inform, educate, and motivate our clinicians and blood bank staff regarding the hospital's commitment and responsibility towards quality and patient safety by reducing the risk of errors, adverse events, and complications. Understanding the importance of framing MSBOS in the future will enable us to ensure better resource allocation for the blood bank. Corrective and preventive actions implemented for various NABH-mandated quality indicators to achieve the benchmark will aid us in initiating several quality improvement projects in the future.
